# LITAF Mediation of Increased TNF-α Secretion from Inflamed Colonic Lamina Propria Macrophages

**DOI:** 10.1371/journal.pone.0025849

**Published:** 2011-09-30

**Authors:** Kristen N. Bushell, Susan E. Leeman, Earl Gillespie, Adam C. Gower, Karen L. Reed, Arthur F. Stucchi, James M. Becker, Salomon Amar

**Affiliations:** 1 Boston University School of Medicine Department of Pharmacology and Experimental Therapeutics, Boston, Massachusetts, United States of America; 2 Boston University Medical Center Department of Surgery, Boston, Massachusetts, United States of America; 3 Boston University Goldman School of Dental Medicine, Center for Anti-Inflammatory Therapeutics, Boston, Massachusetts, United States of America; University Medical Center Freiburg, Germany

## Abstract

Dysregulation of TNF-α in lamina propria macrophages (LPM) is a feature of inflammatory bowel diseases (IBD). LPS-Induced-TNF-Alpha-Factor (LITAF) is a transcription factor that mediates TNF-α expression. To determine whether LITAF participates in the mediation of TNF-α expression in acutely inflamed colonic tissues, we first established the TNBS-induced colonic inflammation model in C57BL/6 mice. LPM were harvested from non-inflamed and inflamed colonic tissue and inflammatory parameters TNF-α and LITAF mRNA and protein levels were measured ex-vivo. LPM from TNBS-treated mice secreted significantly more TNF-α at basal state and in response to LPS than LPM from untreated mice (p<0.05). LITAF mRNA and protein levels were elevated in LPM from TNBS compared with untreated animals and LPS further increased LITAF protein levels in LPM from inflamed tissue (P<0.05). To further confirm the role of LITAF in acutely inflamed colonic tissues, TNBS-induced colonic inflammation was produced in LITAF macrophage specific knockout mice (LITAF mac -/- mice) and compared to wild type (WT) C57BL/6. Twenty four hours following TNBS administration, colonic tissue from LITAF mac -/- mice had less MPO activity and reduced colonic TNF-α mRNA then WT C57BL/6 mice (p<0.05). LPM harvested from LITAF mac -/- secreted significantly less TNF-α in response to LPS than wild type (WT) C57BL/6 (p<0.05). This study provides evidence that LITAF contributes to the regulation of TNF-α in LPM harvested following acute inflammation or LPS treatment paving the way for future work focusing on LITAF inhibitors in the treatment of TNF-α-mediated inflammatory conditions.

## Introduction

Tumor Necrosis Factor alpha (TNF-α) is a cytokine that has a wide variety of functions including immune cell activation and proliferation.[1], [2] TNF-α is produced primarily by macrophages in response to bacterial byproducts such as lipopolysaccharide (LPS). Inflammatory bowel diseases such as Crohn's Disease (CD), are inflammatory disorders characterized by recurring episodes of acute colonic inflammation in the gastrointestinal tract.[3] Clinical and molecular studies have implicated TNF-α as a key mediator in gastrointestinal inflammatory diseases. The excessive production of TNF-α can lead to cytotoxicity and tissue damage associated with inflammation. Anti-TNF-α strategies, including antibodies to TNF-α, have enabled important advances in the medical management of CD. Unfortunately, about 30% of patients remain refractory to current anti-TNF-α antibody therapies,[4], [5], [6] and many more must discontinue use due to adverse effects including infection. Therefore, continued research into new strategies for the therapeutic regulation of TNF-α action is warranted.

TNF-α gene expression is highly regulated by a complex promoter region that contains multiple response elements, including the proinflammatory transcription factor NF-κB.[7] In 1999, Myokai *et al.* reported the isolation and characterization of a transcription factor termed Lipopolysaccharide Induced TNF-Alpha Factor (LITAF), and showed that LITAF plays a role in the regulation of TNF-α gene expression in a human monocyte/macrophage cell line (THP-1).[8], [9] LITAF was shown to specifically bind to the CTCCC sequence on the TNF-α promoter and modulate TNF-α gene expression.[10], [11] Stucchi *et al*. also reported that both LITAF and TNF-α mRNA and protein levels are increased in colonic tissue resected from patients with CD and ulcerative colitis specifically localized to lamina propria macrophages (LPM).[12] The presence of LITAF has been confirmed in mice,[13] and recent evidence has linked increased LITAF mRNA and TNF-α expression levels in a spontaneous genetic mouse model of IBD.[14]

Recently, LITAF mac -/- mice were generated using a Cre/LoxP expression system to specifically target LITAF expression in cells of the monocyte cell lineage.[15] The purpose of the present study was to investigate the role of LITAF in the expression and secretion of TNF-α in LPM harvested from a model of acute inflammation of the colon. The ex vivo characterization of LPM harvested from colonic tissue has led to the documentation of altered properties of LPM following an in vivo inflammatory stimulus. This study also shows that LITAF contributes to the increase in TNF-α secretion in macrophages from inflamed colonic tissue and highlight a potential value of anti-LITAF strategies for the therapeutic management of inflammatory conditions known to be associated with TNF-α such as IBD.

## Materials and Methods

### TNBS Induced Colonic Inflammation

Mice were housed in Association for Assessment and Accreditation for Laboratory Animal Care-approved facilities and protocols were approved by the Boston University Medical Center's Institutional Animal Care and Use Committee. Experiments were conducted in adult, gender-matched male and female C57BL/6 mice obtained from Charles River Laboratories (Wilmington, DE). LITAF mac -/- mice were generated on a C57BL/6 mouse background using the Cre-LoxP system as previously described by Tang et. al. [15] Prior to usage in our experiments, LysMCre-mediated LITAF deletion was confirmed by PCR and Western blot analyses of lysates of the peritoneal macrophages harvested by saline lavage. Treated animals were administered TNBS (*BioChemika*, 1 M in H_2_O) (1.5 mg in 100 µL of 50% Ethanol/water solution) by enema under isoflurane anesthesia. Colonic tissue was dissected at 24–72-hours after TNBS treatment at times associated with acute inflammation in prior studies.[16], [17], [18]

### Assessment of Colonic Inflammation

#### Histology

Colonic tissue was dissected and fixed in 10% formalin prior to being imbedded in paraffin. The paraffin blocks were then cut into 5 µm specimens and fixed to a slide and then stained with hematoxylin and eosin using an automated staining system Sakura Diversified Stainer DRS-601 (Somagen Diagnostics, Edmonton, Alberta). The histological score of each tissue sample was assessed by a blinded observer and assigned a score of 0-4 based on a previously described method [19]. Briefly, a score of 0 was given if there was no change from normal colonic tissue. A score of 1 was given if some epithelial hyperplasia and mononuclear cell infiltrate in a few lesions was observed. A score of 2 was given if the lesions were more extensive and included mucus and goblet cell depletion. A score of 3 was given if the lesions were numerous, extended across large areas of the mucosa, or into the submucosal layers. A score of 4 was given if the lesions observed were more severe and most of the colonic tissue was involved in the inflammation. This includes leukocyte infiltration, severe epithelial hyperplasia, crypt abscesses and ulcers. Five sections per mouse were scored; where possible, a section from the cecum, ascending, transverse, and descending colon and rectum were individually scored. All histopathological sections were reviewed by Dr. Michael O'Brien, MD, MPH GI Pathologist.

#### Myeloperoxidase Activity Assay

Equal weight colonic tissue sections were completely homogenized on ice using a Polytron PT-2100 (*Kinematica, Inc.*, Bohemia, NY) in homogenization buffer (5 mM phosphate buffer, pH 6.0). Samples were then centrifuged at 43,000 x g for 30 minutes at 4°C. Cellular pellets were washed with homogenization buffer and centrifuged again, as above. Following this wash, the pellet was resuspended in sonication buffer (50 mM Phosphate Buffer, pH 6.0 containing 0.5% hexadecyltrimethylammonium bromide) to a final concentration of 100 mg/mL and sonicated (Sonic Dismembrator, Fisher Scientific, Pittsburgh, Pa) on ice for 5, 2-second intervals at full power. Samples were incubated on ice for 20 minutes and then centrifuged for 15 minutes at 12,500 x g at 4°C. To perform the myeloperoxidase (MPO) activity assay, samples and known standards were allowed to react with assay buffer (160 mM phosphate, pH 5.4, 30 mM hydrogen peroxide, 10 mM tetramethylbenzidine in water) for exactly 3 minutes before the addition of the stop buffer (0.66 M sulfuric acid). Absorbance was read in samples and standards at 450 nm and milliunits (mU) of MPO were determined.

#### Lamina Propria Macrophages

Lamina propria macrophages (LPM) were harvested from freshly dissected colonic tissues.[20] Briefly, after the colons were dissected from mice, the fecal contents and mesenteric tissue was removed and the colon tissue was cut lengthwise and extensively rinsed with calcium- and magnesium-free Hank's balanced salt solution (HBSS; Mediatech, Inc., Herndon, VA) and then cut into 5 mm long pieces and added to 50 mL of Digestion Solution I [HBSS with 0.37 mg/mL EDTA and 0.145 mg/mL dithiothreitol (DTT)]. The tissue was then vigorously shaken (110 strokes/min) at 37°C (water bath). After 15 min, the media was replaced with fresh Digestion Solution I (50 mL) for a second incubation. The tissue pieces, which contain the LPM, were rinsed with 50 mL of HBSS, minced into approximately 1 mm pieces and added to 20 mL Digestion Solution II [RPMI 1640 (Sigma-Aldrich, St. Louis, MO), 10% heat-inactivated fetal bovine serum (FBS) (Invitrogen Corporation, Carlsbad, CA), 2.5% Antibiotic-Antimycotic (Mediatech, Inc., Herndon, VA), 400 U/mL collagenase D (Roche Biochemicals, Indianapolis, IN) and 0.1 mg/mL DNase type II (Calbiochem, San Diego, CA)]. The tissue slurry was shaken for 1 hour in a 37°C water bath, and then serially passed through a 1 mm stainless steel sieve followed by 100 µm disposable sieve (BD Biosciences, Franklin Lakes, NJ). Cells in the filtrate were washed twice in HBSS and purified by centrifugation in a 30% percoll (Amersham Biosciences, Piscataway, NJ) solution diluted in complete RPMI 1640. The LPM-enriched pellet, confirmed by cytology to be >98% pure (Diff-Quik and Papanicolaou stain) was resuspended in complete RPMI 1640 media. The LPM were incubated at 37°C with 5% CO2 overnight in the appropriately sized polystyrene dish and non-adherent cells were removed prior to further treatments. Harvested LPM's were washed with HBSS and treated with 1 µg/mL O55:B5 E. coli gel purified LPS (Sigma-Aldrich, St. Louis, MO) in RPMI 1640 media containing 10% heat-inactivated FBS and 2.5% Antibiotic-Antimycotic for 24 hours. This dose is commonly used for treating monocyte/macrophages in culture.[21], [22] Untreated cells served as control cells in every experiment. In some ex vivo experiments LPM were treated with cell signaling inhibitors BAY11-7082 (5 µM) and SB-202190 (50 nM) 1 hour prior to, and during the course of, LPS stimulation. Cells were incubated for the indicated time period before media was collected. Media was pre-cleared by centrifugation (1000xg) and stored at −80°C until further analysis.

### Measurement of TNF-α by ELISA Assay

TNF-α levels in the media were assessed using a mouse TNF-α ELISA set (BD Biosciences, San Diego, CA) according to the manufacturer's instructions. TNF-α concentrations were determined using a standard and values were normalized to total DNA present in the well using DNA quantification kit (Sigma-Aldrich, St. Louis, MO).

### RNA Extraction and Quantitative RT- PCR for LITAF and TNF-α mRNA Levels

Total RNA was isolated from LPM and colon tissue as previously described using the SV Total RNA Isolation System (Promega, Madison, WI).[20] Briefly, primary cells were collected in SV RNA lysis Buffer and added to the provided column according to manufacturer's instructions. The RNA samples were eluted in nuclease-free water and were quantified using a Nanodrop spectrophotometer. Quantitative real-time reverse transcriptase-Polymerase Chain Reaction (qPCR) was used to quantify the mRNA levels of LITAF and TNF-α in isolated LPM. RT-PCR was conducted with the Gene-Amp RNA PCR System (Applied Biosystems, Foster City, CA) as described previously [23] using a DNA Engine Dyad® thermal cycler (Bio-Rad, Hercules, CA). Expression levels were quantified and normalized to the housekeeping gene β-actin mRNA within each sample as an internal control prior to further analysis. In addition, a 15 µL aliquot of the PCR mixture was separated by electrophoresis on a 1.8% agarose gel and stained with ethidium bromide to confirm a single PCR product.

### Western Blotting for LITAF Protein Levels

LPM were washed with HBSS and lysed with M-PER solution (Thermo Scientific, Rockford, IL) containing protease inhibitor cocktail (100x). Lysates were sonicated using a Sonic Dismembrator (Fisher Scientific, Pittsburgh, Pa), for 10 seconds on ice. Total protein content in cleared lysates was determined using BIO-RAD Protein assay (BIO-RAD). Samples were separated using Invitrogen gels (4–12% Bis-Tris 120 V for 1.5 hours) transferred to PVDF membranes using 30 V for 1 hour. LITAF protein levels were detected using a custom made LITAF antibody generated against the c-terminal region of mouse LITAF (Biosynthesis, Inc., Lewisville, TX). This antibody was shown previously to be specific for both human and mouse forms of LITAF. Blots were then stripped and reprobed for the housekeeping protein Actin or GAPDH. Optical density of the bands was determined using Scion image (Scion Corp., Frederick, Maryland). OD for LITAF levels were normalized to the OD of housekeeping proteins.

### Statistical Analysis

All of the data are expressed as means ± SEM and were analyzed by 1-way or 2-way analysis of variance with the Sigma Stat program (SPSS Inc, Chicago, IL). When significant effects (p<0.05) were detected, the differences between specific means were determined by post hoc analysis. In all cases a Dunn test of analysis of variance by ranks followed by the appropriate nonparametric post hoc test was used. Groups were deemed to be significantly different from one another when p<0.05.

## Results

### Evidence that TNBS Induces Colonic Inflammation

**Figure 1:**
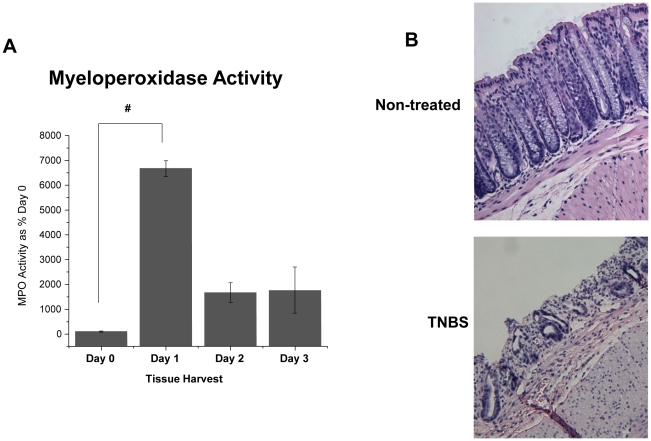
TNBS-induced inflammation in C57BL/6 Mice. Inflammation was induced by TNBS (1.5 mg, in 50% ethanol solution) and animals were monitored for: A) Colonic tissue was collected and assessed for MPO activity (N = 4, except Day 1, N = 3). Data are expressed as % Day 0 Control ± SEM. ^#^ Statistical difference (p<0.001). ***B***) Representative histological images of colonic tissue from C57BL/6 mice 24 hours following TNBS administration showing colonic inflammation (Mag 100×).

Following TNBS administration, colonic tissue levels of MPO activity were significantly increased by 24 hours compared to non-inflamed controls indicating a substantial inflammatory response (Figure 1A) (p<0.001). Consistent with the use of TNBS to induce acute colonic inflammation in C57BL/6 mice, the inflammatory response was markedly reduced on day 2 and 3 compared with day 1. Histological analysis of colonic tissue 24 hours following TNBS administration confirmed the severity of the inflammation and subsequent mucosal damage compared with untreated animals (Figure 1B). Based on these data, all subsequent ex vivo experiments utilized LPM harvested 24 hours after TNBS administration.

### Baseline and LPS-Induced Secretion of TNF-α is Increased in LPM harvested from Inflamed Colons

**Figure 2:**
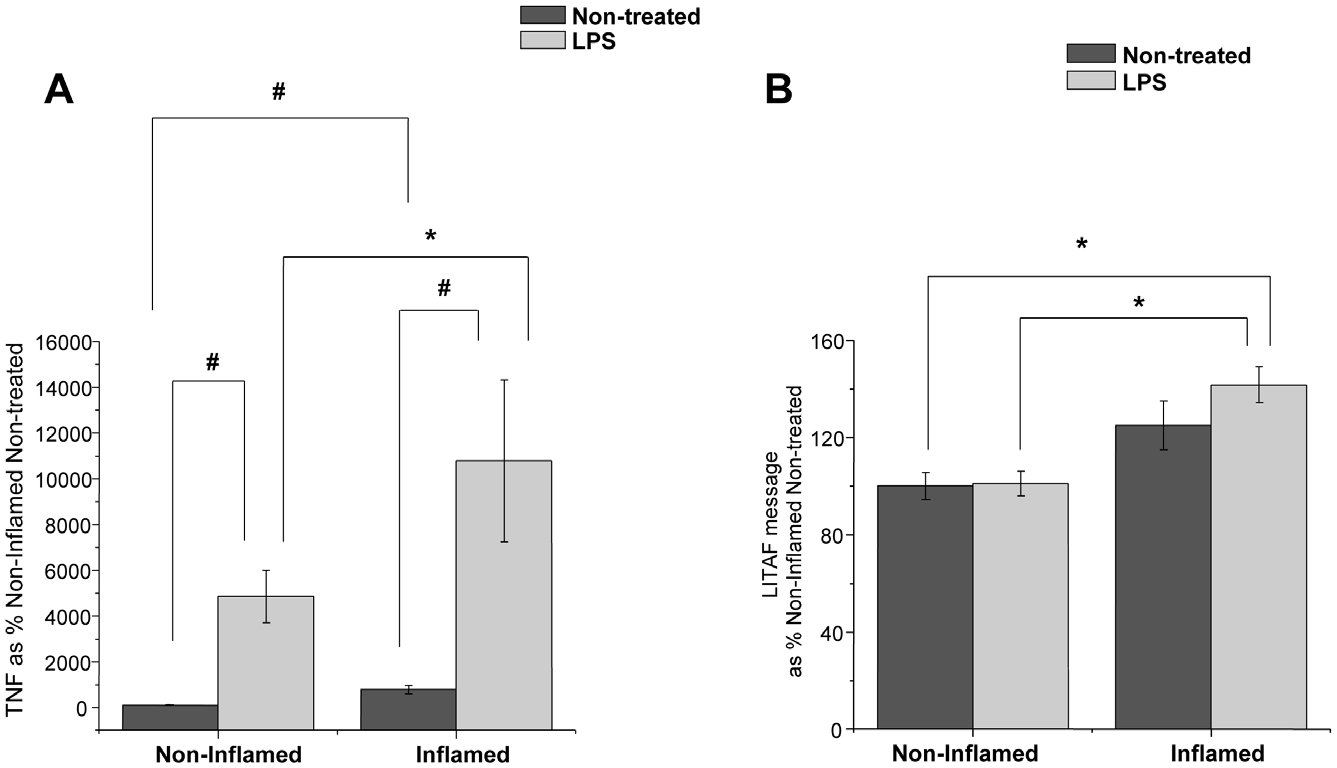
Secreted TNF-α levels and LITAF message are increased in LPM harvested from inflamed colonic tissue compared to LPM harvested from non-inflamed colonic tissue. ***A***) Following TNBS administration, LPM were harvested and stimulated with LPS (1 ug/mL) *ex vivo* (24 hours) and TNF-α secretion was measured (N = 8). Data are presented as % Non-Inflamed Non-treated control ± SEM. * Statistical difference (p<0.05), # statistical difference (p<0.001). B) LITAF message levels are increased in LPM harvested from inflamed colonic tissue following LPS (1 ug/mL) stimulation (1 hour) compared to LPM harvested from non-inflamed colonic tissue (N = 7 for Non-treated, N = 8 LPS treated). Data were normalized to β-Actin message and presented as % Non-Inflamed Non-treated group ± SEM. * Statistical difference (p<0.05).

The baseline secretion of TNF-α into the media was significantly increased in freshly harvested LPM from inflamed colonic tissue from TNBS-treated mice compared to LPM from non-inflamed colonic tissue from non-treated controls (p<0.001) clearly documenting a change in the properties of the LPM in response to the inflammatory stimulus (Figure 2A). After 24 hours of stimulation with LPS (1 ug/mL) ex vivo, TNF-α secretion in LPM harvested from both inflamed and non-inflamed colonic tissue was significantly increased (p<0.05) compared with non-treated controls, but the TNF-α secretion from LPM harvested from inflamed colonic tissue was >2-fold greater than the TNF-α secretion from LPM harvested from non-inflamed animals (p<0.05) (Figure 2A). These data show that the properties of the LPM have been altered by the *in vivo* TNBS inflammatory stimulus such that the ex vivo response to LPS is significantly greater.

### LPS Increases LITAF mRNA and Protein in LPM Harvested from Inflamed Colonic Tissue

**Figure 3:**
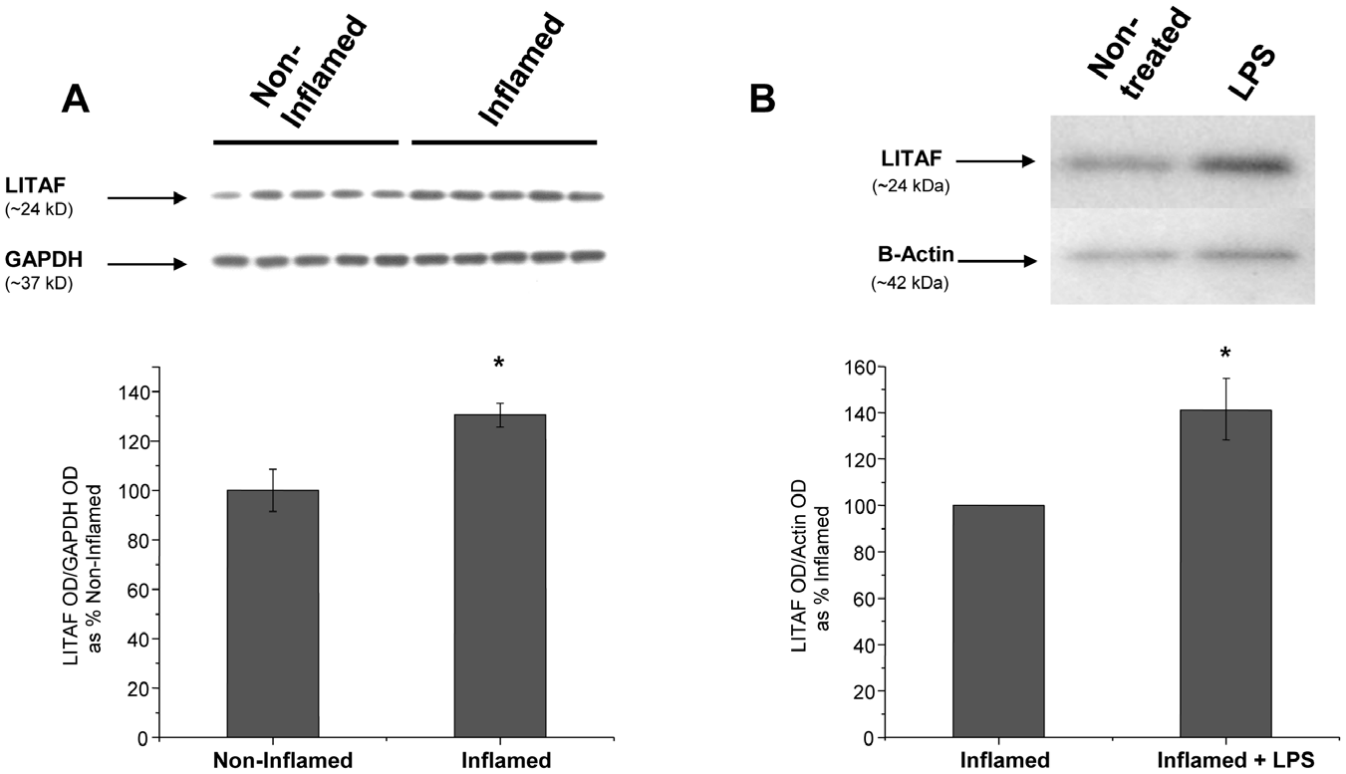
LITAF protein levels are increased in LPM harvested from inflamed colonic tissue and further increased in inflamed LPM following LPS (1 ug/mL) stimulation (N = 5). ***A***) Western blot of LITAF levels and GAPDH levels in freshly harvested LPM from Non-Inflamed and Inflamed colonic tissue. The graph shows the optical density (OD) of LITAF levels normalized to GAPDH levels. * Statistical difference from Non-Inflamed group (p<0.05). B) Western blot of LITAF levels and β-Actin levels in LPM harvested from inflamed colonic tissue stimulated with LPS ex vivo compared to LPM harvested from inflamed colonic tissue, but not treated with LPS (Non-treated). The graph show OD of LITAF levels normalized to β-Actin levels. * Statistical difference (p<0.05).

LITAF mRNA levels were significantly (p<0.05) increased in LPM harvested from inflamed colonic tissue and stimulated with LPS compared to LPM harvested from non-inflamed colonic tissue (Figure 2B). Although no statistical differences were detected between LITAF mRNA levels in LPM isolated from inflamed tissues compared to LPM harvested from normal tissues, subsequent LPS treatment of LPM isolated from inflamed tissues did modestly but significantly increase (p<0.05) LITAF mRNA levels (Figure 2B). LITAF protein levels, determined by western blot, were also increased 30% (p<0.05) in LPM harvested from inflamed colonic tissue compared to LPM harvested from non-inflamed colonic tissue (Figure 3A). In LPM harvested from inflamed colonic tissue, *ex vivo* stimulation with LPS induced a 40% increase (p<0.05) in LITAF protein compared with non-treated LPM harvested from inflamed colonic tissue (Figure 3B). The increase in LITAF levels following an inflammatory stimulus supports the possibility that LITAF plays a role in the increased TNF-α secretion in these cells.

### LPS-Induced Secretion of TNF-α is inhibited by Inhibitors of p38 and NF-kB from LPM Harvested from Inflamed Colons

**Figure 4:**
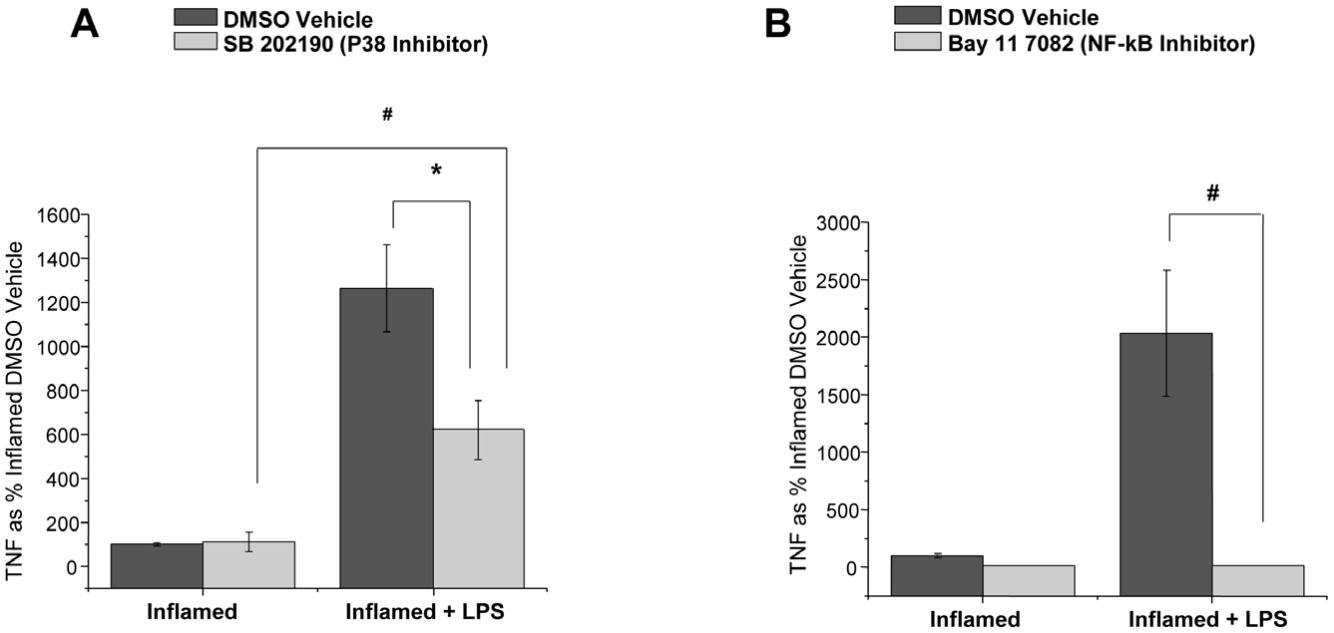
Secreted TNF-α levels from LPM harvested from inflamed colonic tissue are reduced with the administration of signaling inhibitors. ***A***) Secreted TNF-α from LPM harvested from inflamed colonic tissue are reduced when administered p38 inhibitor (SB-202190) prior to, and throughout LPS (1 ug/mL) stimulation (24 hours) (N = 8). Data are expressed as % Inflamed DMSO vehicle ±SEM. * Statistical difference (p<0.05), ^#^ statistical difference (p<0.001). B) Secreted TNF-α from LPM harvested from inflamed colonic tissue are reduced when administered NF-κB inhibitor (BAY 11-7082) prior to, and throughout LPS (1 ug/mL) stimulation (24 hours) (N = 6). Data are expressed as % Inflamed DMSO Vehicle group ± SEM. ^#^ Statistical difference (p<0.001).

In the context of the potential importance of anti-LITAF therapeutic approaches we investigated whether an inhibitor of p38 would reduce the elevated TNF-α response in LPM harvested from inflamed colonic tissue since p38 signaling pathway is known to be involved in LITAF activation [15]. LPM treated with the p38 inhibitor, SB-202190-treated LPM had a significant (p<0.05) reduction in TNF-α secretion compared to LPS stimulated DMSO vehicle controls (Figure 4A). SB-202190 had no effect on TNF-α secretion from LPM not stimulated by LPS. Additionally, since LITAF is known to bind on sites other than consensus NF-κB sequences on the TNF-α promoter, we looked for the role of NF-κB inhibitors in this ex-vivo model. LPM harvested from inflamed colonic tissue were treated with a NF-κB inhibitor, BAY11-7082, prior to and during LPS stimulation have significantly (p<0.001) reduced TNF-α secretion compared to LPS stimulated DMSO vehicle controls (Figure 4B). BAY11-7082 had no effect on TNF-α secretion from LPM not stimulated by LPS. These results support the conclusion that TNF-α secretion in LPM harvested from inflamed colonic tissue is mediated by both NF-κB and p38 transcriptional pathways.

### LPS-Induced Secretion of TNF-α is less in LPM Harvested from Inflamed Colons of LITAF mac -/- Mice

**Figure 5:**
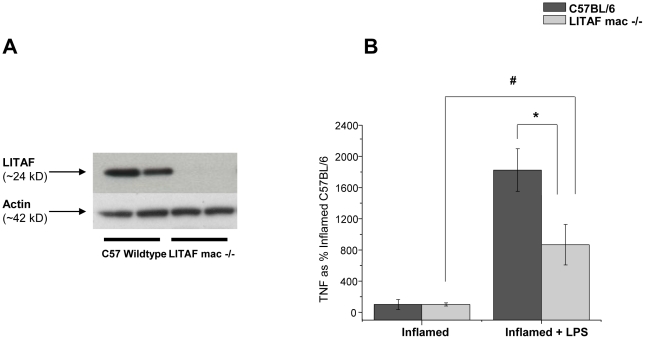
LPM harvested from LITAF macrophage specific knockout mice (LITAF mac -/- mice) produce less TNF-α than LPM harvested from C57BL/6 mice. ***A***) Western blot of LITAF and Actin confirming the knockout of LITAF protein in peritoneal macrophages harvested from LITAF mac -/- and C57BL/6 mice. ***B***) TNF-α secretion from inflamed LPM isolated from colonic tissues of LITAF mac -/- (N = 6) or wildtype C57BL/6 (N = 12) following LPS stimulation (24 hours). Data are expressed as % Inflamed C57BL/6 group ± SEM. * Statistical difference (p<0.05), # statistical difference (p<0.001).

The deletion of the LITAF gene in LITAF mac -/- was confirmed by western blot of peritoneal macrophages collected by peritoneal lavage at time of necropsy and compared to peritoneal macrophages harvested from a C57BL/6 wildtype mice (Figure 5A). Peritoneal macrophages were used in lieu of LPM from the LITAF mac -/- animals to confirm the absence of LITAF due to the limited number of cells obtained from harvesting LPM macrophages. LITAF deficient LPM harvested from LITAF mac -/- mice following TNBS administration secrete significantly less TNF-α (p<0.05) following LPS stimulation ex vivo, than LPM from wildtype C57BL/6 mice (Figure 5B). These data provide evidence that LITAF mediates the LPS-induced TNF-α secretion from LPM harvested from inflamed colonic tissue.

### Bodyweight and Colonic Inflammatory Response to TNBS are less in LITAF mac -/- Mice

**Figure 6:**
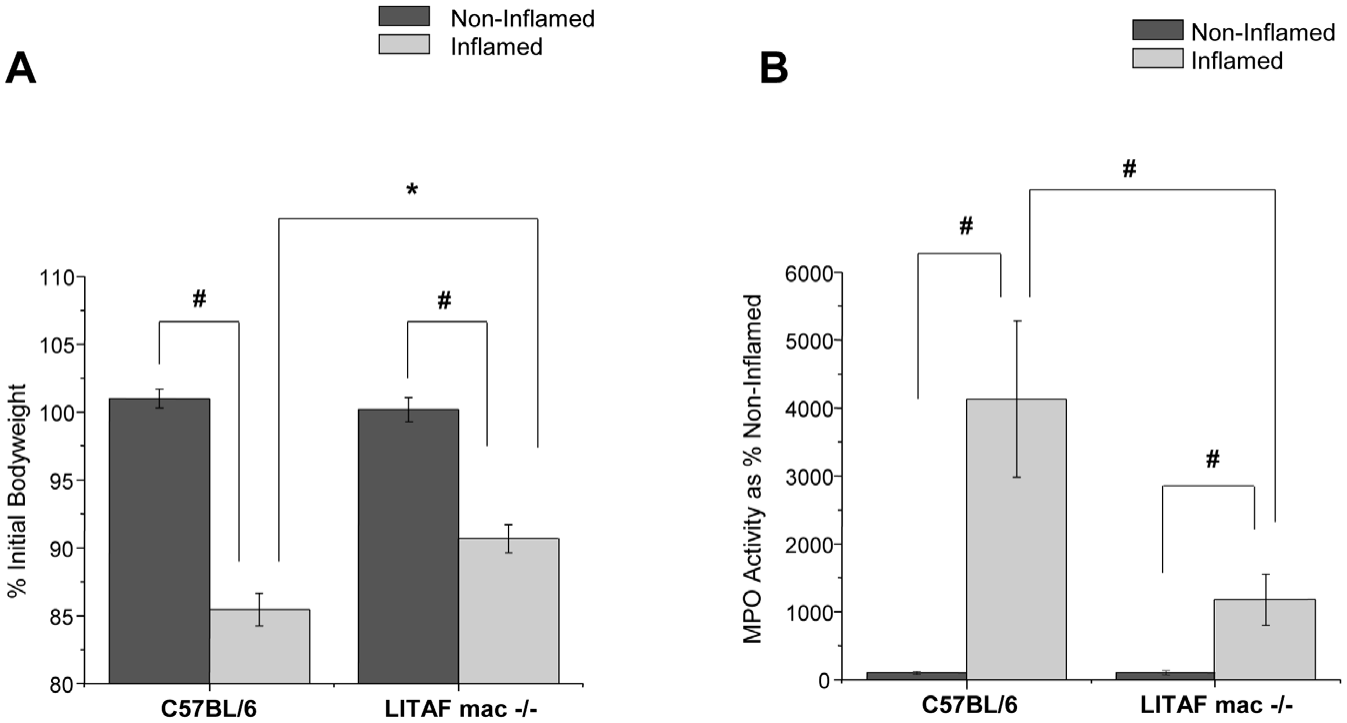
LITAF macrophage specific knockout mice (LITAF mac -/-) show reduced changes in bodyweight and MPO following TNBS administration. ***A***) Following TNBS (3.0 mg) administration to LITAF mac -/- mice (N = 6) and C57BL/6 mice (N = 6), bodyweights were measured 24 hours later. Data are expressed as % initial (day 0) bodyweight. * Statistical difference (p<0.05), # statistical difference (p<0.001). B) Following TNBS (3.0 mg) administration to LITAF mac -/- mice (N = 6) and C57BL/6 mice (N = 6), colonic tissue was collected and assessed for MPO activity. Data are expressed as % Non-inflamed MPO activity. ^#^ Statistical difference (p<0.001).

**Figure 7:**
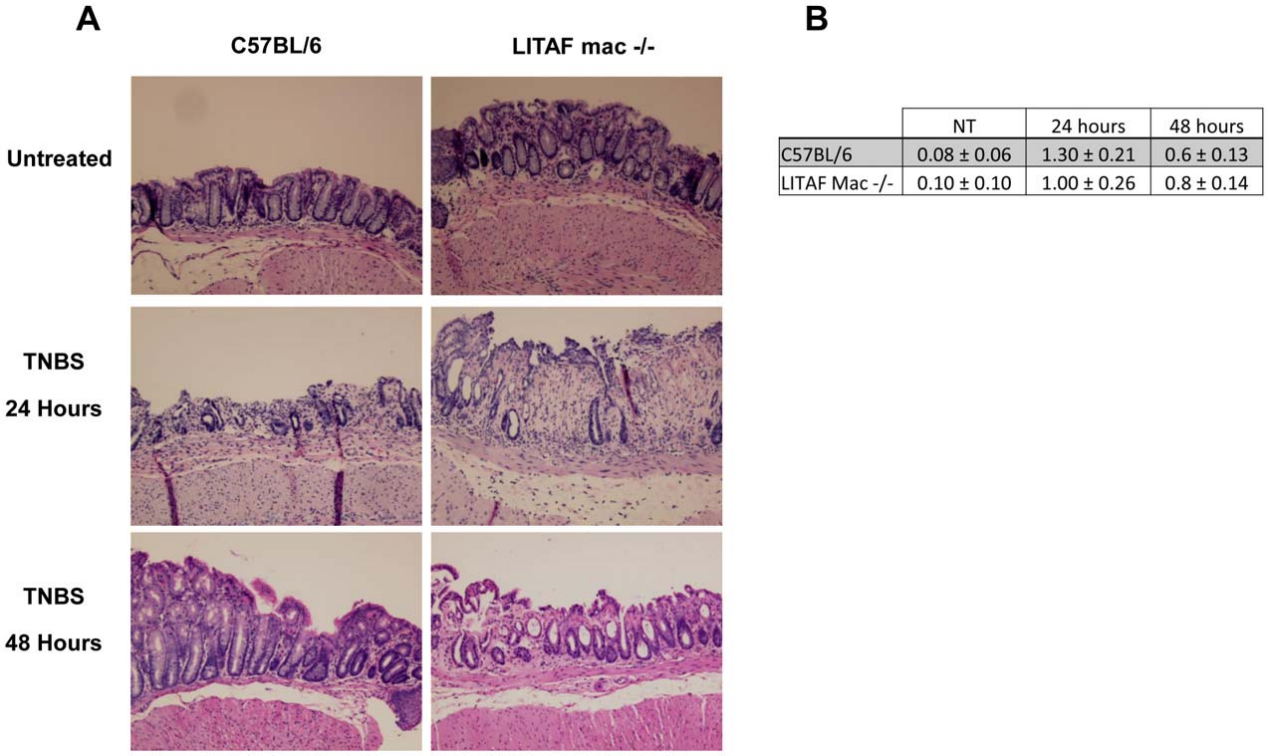
TNBS-induced histological damage is not reduced in LITAF mac -/- mice. ***A***) Representative histological images of colonic inflammation: Untreated Control; TNBS 24 hours; and, TNBS 48 hours following TNBS administration. (N = 3) images (Mag 100×). ***B***) Histological score (0–4) ± SEM (N = 5) of colonic tissue 24 and 48 hours following TNBS administration.

**Figure 8:**
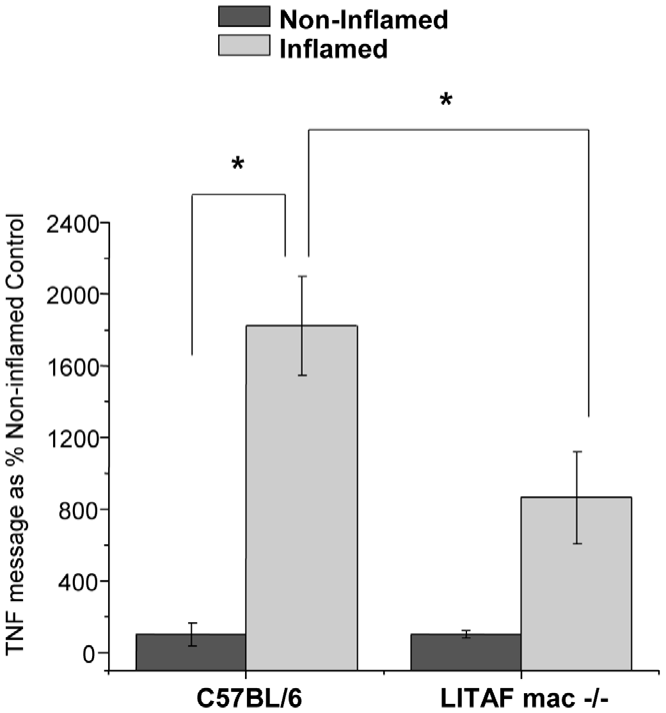
TNF-α message levels from inflamed colonic tissue harvested from LITAF macrophage specific mice (LITAF mac -/- mice) is reduced compared to C57BL/6 mice. TNF-α message levels were measured in colonic tissue following TNBS administration (0.3 mg) for 24 hrs in LITAF mac -/- mice (N = 6) and C57BL/6 mice (N = 12). Data are expressed as % Non-Inflamed control ± SEM. * Statistical difference (p<0.05).

In addition to the reduced TNF-α secretory response in LPM harvested from the inflamed colons of LITAF mac -/- mice, there were also markedly reduced inflammatory responses to TNBS administration *in vivo*. LITAF mac -/- mice lost significantly (P<0.05) less body weight in response to a 24 hours inflammatory challenge with TNBS than did their counterpart wildtype C57BL/6 mice (Figure 6A). Furthermore, following TNBS administration, colonic samples harvested from LITAF mac -/- mice showed significantly (p<0.001) reduced MPO levels compared to the inflamed colons from C57BL/6 mice (Figure 6B). Interestingly, histological samples taken from LITAF mac -/- mice at 24 and 48 hours following TNBS administration do not show reduced colonic inflammation overall compared to control C57BL/6 mice (Figure 7A). Scoring of the histological samples based on microscopic changes to epithelial layer, crypt depth, edema and neutrophil infiltration showed no significant overall changes between C57BL/6 and LITAF mac -/- mice at 24 or 48 hours following TNBS administration (Figure 7B). Indeed, while the majority of colonic tissue in both wild-type and knock-out animals shows only mild inflammation, there is more mucosal damage in the LITAF mac -/- mice where severe ulceration occurs, with more neutrophil infiltration, crypt damage, and thickened or necrotic mucosa due to edema 24 hours following TNBS administration. At 48 hours following TNBS administration, the C57BL/6 mice show more signs of tissue repair while LITAF mac-/- mice still have signs of mucosal and crypt damage in the ulcerated area. However, colonic samples from LITAF mac -/- mice following TNBS administration have significantly (p<0.05) reduced TNF-α mRNA levels compared to inflamed colonic samples from C57BL/6 mice (Figure 8). These results support that LITAF mediates TNF-α expression in colonic inflammation but indicates that TNBS-induced colonic inflammation is a multi-factorial process mediated by many inflammatory signaling pathways.

## Discussion

The present study demonstrates that LITAF plays an important role in the regulation of TNF-α secretion in the inflamed colon. Following a TNBS inflammatory stimulus, LPM harvested from acutely inflamed colonic tissue secreted higher basal levels of TNF-α compared to LPM harvested from non-inflamed tissue; LPS was found to further increase this TNF-α secretion. These findings are further strengthened by our data demonstrating that TNF-α secretion is significantly reduced in LPM harvested from the inflamed colons of LITAF mac -/- knockout mice in which the LITAF gene has been selectively deleted from macrophages. The present data constitute the first demonstration that LITAF mRNA expression and protein levels are increased in LPM isolated from inflamed colonic tissues compared to non-inflamed colonic tissues, strongly supporting the likelihood that LITAF mediates in part the increase in TNF-α secretion. Collectively the present data implicate LITAF in the regulation of TNF-α secretion.

In order to compare macrophage TNF-α secretion responses following colonic inflammation with and without LITAF, it was necessary to use C57BL/6 mice because LITAF mac -/- mice were generated in same strain.[15] Although C57BL/6 mice are generally resistant to TNBS-induced inflammation[17] it has been shown that this mouse strain does exhibit a quantifiable inflammation during the first 24–48 hours following TNBS administration.[24] This feature of milder degree of inflammation permitted the isolation of viable LPM for analyses *ex vivo*. LPM harvested from inflamed colonic tissue have advantages over monocyte/macrophage cell lines, such as THP-1 cells, or macrophages specific to other organs since the specific phenotype is dictated by the microenvironment in which a macrophage matures.[25] Primary isolation of LMP also allows for the *in vivo* activation of the specific cells which under non-inflamed conditions show a hyporesponsiveness to LPS.[26] Given the short duration of the colon inflammation studied (24 hours), it is highly probable that the LPM harvested are resident LPM and are not newly recruited monocytes and macrophages whose recruitment to inflamed tissue has been determined to occur from 2 to 10 days following injury.[27]

Inflammatory signaling in macrophages begins with activation of the Toll like Receptor 4 (TLR4) which leads to activation of Mitogen Activated Protein Kinase (MAPK) pathways, including p38.[3] Downstream targets of p38 include numerous transcriptional regulators, including AP-1 and C/EBP-1.[28] In addition, p38 has been shown to activate LITAF with no significant changes of TNF-α protein levels in macLITAF^−/−^ cells either treated with LPS alone or cotreated with SB203580 plus LPS.[15] Inhibitors of p38 were used in this *ex vivo* system to determine if inhibition could reduce TNF-α secretion from LPM harvested from inflamed colonic tissue. A commonly used p38 specific inhibitor SB-202190 [29], [30] partially reduces the TNF-α response in LPM harvested from inflamed colonic tissue following LPS stimulation. While p38 inhibition likely leads to many downstream effects, the observed reduction in TNF-α secretion is consistent with an inhibition of the LITAF contribution to TNF-α secretion.

Additionally, TLR4 is known to activate the NF-κB pathway through the ubiquitination and degradation of IκB. IκB degradation allows for the translocation of the p65 and p50 heterodimeric unit to the nucleus where transcription of inflammatory genes, including TNF-α, is induced.[31], [32] In this *ex vivo* system, inhibitors of NF-κB signaling also caused a reduction in TNF-α secretion in response to LPS stimulation in LPM harvested from inflamed colonic tissue. This is not surprising given that NF-κB is a ubiquitous transcription factor that regulates over 300 genes, including TNF-α, many involved in inflammation and cancer.[32], [33], [34] The human TNF-α promoter region contains multiple NF-κB consensus sequences, in addition to consensus sequences for many other transcription factors, including SP-1 and Erg1,[35] NF-AT,[36] AP-1 and AP-2,[37] CREB/ATF,[38] and Ets.[39] Although not definitive, taken together, our data lend support to our hypothesis that the increased TNF-α response in LPM harvested from inflamed colonic tissue may stem from multiple pathways including NF-κB, LITAF and p38 MAPK pathways.

The most direct evidence that LITAF is associated with the capacity of LPM to secrete TNF-α comes from the use of LITAF deficient LPM in these studies. Using LPM harvested from LITAF mac -/- mice following TNBS induced inflammation, the data supports the conclusion that the deletion of the LITAF gene leads to a significant reduction in TNF-α secretion in response to LPS stimulation compared to wildtype controls. Since differences in TLR4 mRNA levels were not found between peritoneal macrophages derived from C57BL/6 and LITAF mac -/- mice (data not shown) it very likely that this process is not dependent on changes in TLR4. In this system, the remaining TNF-α signal that we have observed in LITAF deficient macrophages is likely due to the remaining proinflammatory pathways including NF-κB.

LITAF mac -/- mice exhibit reduced bodyweight loss, have lower MPO levels and, on examination of their colonic tissue, express less TNF-α mRNA as compared to C57BL/6 mice. Despite the apparent reduction in the acute TNBS-induced inflammatory response, LITAF mac-/- mice do not exhibit a reduction in the overall histological damage to the colonic tissue following 24 or 48 hours after TNBS administration. These findings suggest that the animals deficient in LITAF have a delayed healing response compared to C57BL/6 mice. This may be due, in part, to the rapid and strong inflammatory stimulus that TNBS administration imparts when administered rectally. Another explanation for this result may be related to a beneficial dysregulation of the inflammatory response in LITAF mac -/- animals.[15], [40] Indeed LITAF mac -/- mice have been shown to have reduced levels of many inflammatory cytokines and chemokines, when administered a sublethal dose of LPS. Compared to the early surge of serum inflammatory cytokines seen in wildtype mice, LITAF mac -/- mice show a delayed increase of proinflammatory cytokines, and a persistent increase of anti-inflammatory cytokines illustrating that the absence of LITAF leads to a beneficial temporal dysregulation of the inflammatory response.[15], [40] The histological inflammatory response observed 24 hours after the acute inflammatory stimulus of TNBS in LITAF mac -/- mice may just reflect a delay of inflammation consistent with the previous report [15], [40].

Interestingly, while making synthetic derivatives of the lactone kavain, Pollastri *et al.* identified an acyclic derivative ((*E*)-5-biphenyl-4-yl-5-hydroxy-3-methoxy-pent-2-enoic acid methyl ester) and showed this compound to suppress LITAF levels while simultaneously reducing TNF-α secretion from macrophages stimulated with LPS.[41] Future studies may lead to optimization of this compound and may increase our understanding of the feasibility of the use of kava compounds to specifically inhibit LITAF and further explore anti-LITAF strategies as a potential treatment for inflammatory diseases known to be associated with increased TNF-α.

In summary, endogenous colonic LPMs increased their base line and LPS mediated TNF-α secretion by a LITAF dependant pathway when harvested from acutely inflamed colonic tissue suggesting the usefulness of anti-LITAF drug development for the treatment ulcerative colitis. We propose that LITAF plays a substantial role in TNF-α secretion from LMP isolated from an acutely inflamed colon and represents an alternative pathway to modulation of the TNF-α component of the inflammatory response. A better understanding of the role that LITAF signaling plays in regulating TNF-α gene expression in the LPM of the inflamed colon may provide alternative targets for therapeutic interventions not only in IBD, but other chronic inflammatory diseases exacerbated by elevated TNF-α levels.
